# Lead Affects Vitamin D Metabolism in Rats

**DOI:** 10.3390/nu10030264

**Published:** 2018-02-26

**Authors:** Abdur Rahman, Ameena A. Al-Awadi, Khalid M. Khan

**Affiliations:** 1 Department of Food Science and Nutrition, College of Life Sciences, Kuwait University, P.O. Box 5969, Safat 13060, Kuwait; alawadi.cfw@gmail.com; 2 Department of Anatomy, Faculty of Medicine, Kuwait University, P.O. Box 24923, Safat 13110, Kuwait; khalid.khan@hsc.edu.kw

**Keywords:** lead, vitamin D, 25-hydroxy vitamin D, 1,25-dihydroxy vitamin D, 25-hydroxylase, 1-α-hydroxylase, vitamin D receptor, rats

## Abstract

A negative association between blood lead and vitamin D concentrations has been reported, however, experimental data on the effect of lead (Pb) on vitamin D metabolism is scarce. We investigated the effects of Pb on serum vitamin D metabolites, vitamin D activating enzymes and vitamin D receptor (VDR) in rats. Newborn Wistar rat pups were exposed to 0.2% Pb-acetate via their dams’ drinking water from post-natal day (PND) 1 to 21 and directly in drinking water until PND30. Serum 25-hydroxyvitamin D was analyzed with LC-MS/MS and 1,25-dihydroxyvitamin D with an immunoassay. Tissue expression of vitamin D activating enzymes and VDR were measured by Western blot and immunohistochemistry. Serum 25-hydroxyvitamin D was significantly decreased at both PND21 and PND30, whereas 1,25-dihydroxyvitamin D was decreased (*p* < 0.05) only at PND21 in the Pb-exposed rats. Expression of renal 1-α-hydroxylase was decreased by Pb only at PND21 (*p* < 0.05) but the brain 1-α-hydroxylase was not affected. Hepatic 25-hydroxylase expression was significantly decreased at PND21 but significantly increased at PND30 by Pb exposure. VDR expression in the brain was increased at both PND21 and PND30 (*p* < 0.05). These results suggest that Pb interferes with vitamin D metabolism by affecting the expression of its metabolizing enzymes.

## 1. Introduction

Vitamin D has been implicated in several physiological functions that extend beyond its classical effects on bone mineralization [1]. One of the recent emerging roles of vitamin D is its involvement in maintaining the health and development of both fetal and adult brain [2,3]. Vitamin D receptors (VDR) and metabolizing enzymes (hepatic 25-hydroxylase and renal 1α-hydroxylase) have been found in several human brain areas [4] which are involved in planning, processing and formation of new memories [4,5].

Lead (Pb) is a known neurotoxic heavy metal [6,7,8] that is ubiquitously present in our environment and is still being used in various industries [9,10]. With the development of modern building structures, transportation and industry, Vitamin D deficiency (VDD) as well as Pb poisoning have become more prevalent at global level, resulting in serious problems especially among children [11,12,13,14,15]. The reported neurological deficits from childhood Pb toxicity [7] have led to the progressive decline in the definition of Pb poisoning reference level from 60 μg/dL to 5 μg/dL [16,17]. Yet, there is no safe level of Pb exposure for children [18,19].

It has been suggested that high Pb exposure is one of the potential risk factor of VDD [20,21]. Studies of children with high Pb exposure demonstrated a significant negative association between 1,25-dihydroxyvitamin D [1,25(OH)_2_D] concentration and blood Pb level (BPbL) [22,23,24,25]. Children whose BPbLs were in the range of 33–55 μg/dL showed a significant reduction in serum 1,25(OH)_2_D concentration. The decrease in serum 1,25(OH)_2_D was even more pronounced in children with BPbL of >62 μg/dL, suggesting a dose-dependent effect of Pb on vitamin D status [24]. Data from animal studies have shown that Pb ingestion decreased serum concentration of 1,25(OH)_2_D and blocked vitamin D-dependent intestinal calcium transport in rats [26]. High BPbL has been suggested to cause disruption of the renal hydroxylation of 25-hydroxyvitamin D [25(OH)D] by 1-α-hydroxylase to produce the active form of vitamin D; 1,25(OH)_2_D [27]. Consequently, reduction of serum 1,25(OH)_2_D synthesis induced by Pb toxicity is accompanied with reduced calcium levels and increased serum parathyroid hormone (PTH) levels. Thus, Pb interferes with various vitamin D functions involved in calcium balance and metabolism in various tissues and organs [28,29,30,31]. However, the mechanism(s) of these effects have not been elucidated. This study was thus conducted to investigate the effect of Pb exposure on (1) serum vitamin D metabolites levels; 25(OH)D and 1,25(OH)_2_D; (2) the expression of vitamin D metabolizing enzymes; 25-hydroxylase in the liver and 1-α-hydroxylase in the kidneys and brain and (3) the expression of VDR in brain of Wistar rat pups exposed to Pb during their early postnatal life.

## 2. Materials and Methods

### 2.1. Pb Exposure Protocol

Wistar rats were used in this study. Animal exposure and handling was according to the approved protocol of Institutional Animal Care and Use Committee of Kuwait University. Animals were housed at constant temperature (21 ± 2 °C) and a relative humidity, under a 12-h light/dark cycle (lights on from 700 to 1900 h) with free access to food and water. Ten females were mated; eight of them became pregnant. Pregnancy was confirmed with swab test of vaginal smear. At birth pups were randomly culled to 7–8 per dam. Pups in the experimental group were exposed to 0.2% Pb acetate via their dams’ drinking water from postnatal day (PND) 1 to PND21 and directly through drinking water until PND30. The control group were given regular tap water. Pb concentration in the tap water was below the detection limit of the assay. The number of pups in the experimental (Pb-exposed) and control groups were 37 and 38, respectively.

### 2.2. Collection of Blood and Tissue Samples

Half of the pups from each group were euthanized with CO_2_, weighed and decapitated at PND21 and the rest at PND30. Blood was drawn from the right ventricle. 100 μL whole blood was aliquoted and stored in the refrigerator (at 4 °C) for Pb analysis. The remaining blood was centrifuged at 12,000× *g* for 15 min to obtain serum for vitamin D metabolites analysis. Serum samples were stored at −80 °C until analysis. Samples were protected from light throughout processing and storage. Cerebral hemispheres, liver and kidneys were dissected out, weighed, frozen in liquid nitrogen and stored at −80 °C until analyses. At the end of the study, dams were euthanized with CO_2_.

### 2.3. Pb Analysis

Pb concentrations in the digested blood samples were quantified with inductively coupled plasma-optical emission spectrometer (ICP-OES). For digestion, whole blood samples (100 uL) were digested by overnight incubation in a mixture of 1 mL nitric acid (ultra-pure) and 1.5 mL perchloric acid, followed by heating (to 50 °C) for 30 min and boiling (at 90 °C) for 15 min. While heating, nitric acid was added gradually to each sample, as needed until the solution turned clear. Nitric acid was evaporated under the fume hood and the dried residue was dissolved in 3 mL of deionized water. Five standards of Pb solutions were prepared with a range of 0, 0.01, 0.05, 0.1 and 0.5 mg/L from standard stock solution by dilution with deionized water.

The system used for Pb analysis was a computer-controlled sequential ICP-OES spectrometer covering a spectral wavelengths range of 160–800 nm (iCAP 6500 ICP-OES; Thermo Fisher Scientific Inc., Cambridge, UK) with 1Kw of RF Power, pump speed of 15 rpm and gas flows of 1.5 L/min for the plasma, 0.5 L/min for the auxiliary flow and below 1 L/min for the nebulizer pressure. A 60-s washing time was performed between each sample. A wavelength of 220.3 was used for Pb detection using the Optimize Source function of Thermo Scientific iTEVA Software (Cat. No. 8499 400 30001, Thermo Fisher Scientific Inc., Cambridge, UK).

### 2.4. Vitamin D Metabolites Analysis

Serum 25(OH)D was analyzed in CAP-accredited laboratory by liquid chromatography-tandem mass spectrometry (LC-MS/MS) as described by Al-Harbi et al. [32], using the commercially available kits from Chromsystems (Cat. #2000/1000/F; Chromsystems Instruments & Chemicals GmbH, Grafelfing, Germany) according to the manufacturer’s instructions. Precision and accuracy of analysis were monitored by including control samples (*MassCheck* controls). The intra-assay and inter-assay CV were 3.7% and 6.0%, respectively. Serum 1,25(OH)_2_D was analyzed by the commercially available Diasorin kit (Cat. # 310981, DiaSorin Inc., Stillwater, MN, USA), using the LIASON, DiaSorin system.

### 2.5. Western Blotting

The expression of 25-hydroxylase, 1-α-hydroxylase and VDR were analyzed by Western blot. Tissue samples were homogenized in RIPA buffer (50 mM Tris, pH 7.4, 150 mM NaCl, 1% NP-40, 5 mM EDTA, 0.5% sodium deoxycholate, 0.1% SDS, 50 nM NaF) and protease inhibitor cocktail (Roche Diagnostic, Castle Hill, NSW, Australia). Concentration of protein in tissue samples were measured by Bradford method. Samples were stored at −80 °C until used.

An aliquot of tissue lysate (20 μg protein) was resolved by electrophoresis on 10% SDS-PAGE (NuPAGE, Invitrogen, Carlsbad, CA, USA). Proteins were transferred onto PVDF membranes and the membranes were blocked with 5% powdered milk in TBS for 1 h and incubated with primary antibodies (Table 1) overnight at 4 °C. The dilution factor for all the primary antibodies was 1:1000. The membranes were washed with TBS-Tween 3× (5 min each) and 2× with TBS only. Afterwards membranes were incubated with the HPR-conjugated secondary antibody for 2 h at room temperature, washed as above and developed using ECL kit (GE Healthcare Life Sciences, Westborough, MA, USA). Beta-actin was used as loading control. Blots were scanned and quantification of band density was measured by using Syngene gene tools software (Gene Tools Image Analysis, Philomath, OR, USA).

### 2.6. Immunohistochemistry

The specificity of antibodies for 25-Hydroxylase, 1-α-hydroxylase and VDR was tested by Western blot before immunohistochemistry. Animals were euthanized with CO_2_ and perfused transcardially with normal saline followed by a fixative that consisted of 4% paraformaldehyde and 0.1% glutaraldehyde in 100 mM phosphate buffer, pH 7.4 at 4 °C. Brain, liver and kidneys were collected and transferred to a cold fixative for 24 h at 4 °C. Tissues were washed overnight in cold phosphate buffer, then dehydrated in graded ethanol, cleared in xylene and embedded in paraffin. Sections (8 μm) were cut using rotary microtome and two sections were mounted on each slide.

Sections were de-paraffinized and rehydrated at room temperature. All sections were quenched for free aldehyde groups and endogenous peroxidase activity with 3% H_2_O_2_ in water and 0.1% sodium borohydride and 50 mM glycine in PBS; respectively. Sections were washed with PBS three times and incubated in 2% normal serum for 1 h at room temperature (25 °C) to block nonspecific binding and kept overnight (for 12–16 h at 4 °C) in primary antibody (Table 1). Next day, sections were washed with PBS three times (5 min each) and incubated in biotin-conjugated secondary antibody for 90 min followed by incubation in streptavidin peroxidase solution for 30 min. Reactive sites were visualized with diaminobenzidine as a chromogen. One section on each slide was counterstained with hematoxylin prior to mounting. Sections that were incubated in normal serum (instead of the primary antibody) served as negative controls.

### 2.7. Statistical Analysis

Means of the data for each group (Pb-exposed and control) were compared by Student t-test for two independent samples with unequal variances. The association of blood Pb with 25(OH)D and 1,25(OH)_2_D was evaluated by linear regression analysis. Data were presented, as mean ± SD and the significance level of difference was set at *p* < 0.05. Data were analyzed by SPSS for Windows version 23 (SPSS Inc., Chicago, IL, USA).

## 3. Results

Pb exposure did not affect the body weight of pups either at PND21 or PND30. Weights of brain, liver and kidneys were also not significantly affected (*p* > 0.05) by Pb-exposure either at PND21 or PND30 (Table 2). Mean BPbL in the Pb-exposed rats was significantly increased (*p* < 0.0001) at both PND21 and PND30 (12.3 and 22.7 μg/dL; respectively) compared to the respective control groups (2.2 and 3.3 μg/dL at PND21 and PND30, respectively) (Table 3).

### 3.1. Effect of Pb Exposure on Serum Vitamin D Metabolites

**Figure 1:**
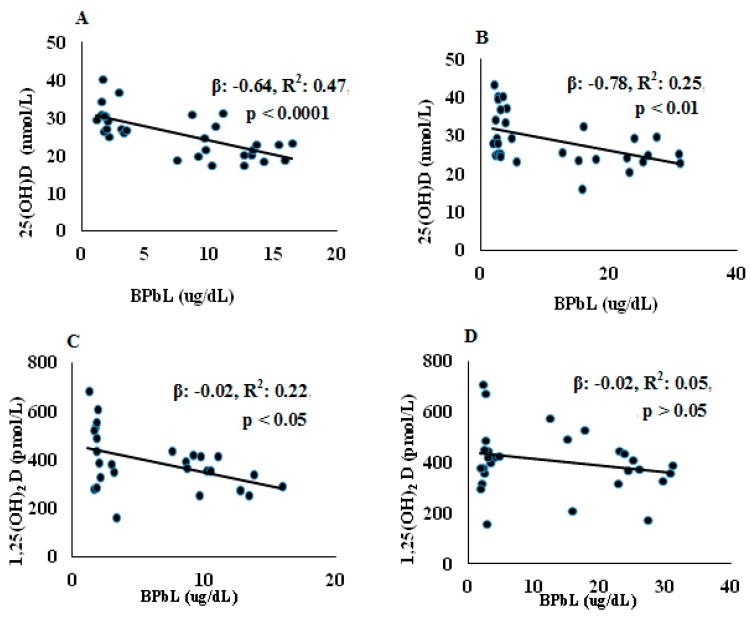
Association between BPbL and VD metabolites in control and Pb-exposed Wistar rat pups. Linear regression analysis was conducted between BPbL (µg/dL) and (**A**) 25(OH)D (nmol/L) in PND21 rats (*n* = 39); (**B**) 25(OH)D (nmol/L) in PND30 rats (*n* = 36); (**C**) 1,25(OH)_2_D (pmol/L) in PND21 rats (*n* = 39); (**D**) 1,25(OH)_2_D (pmol/L) in PND30 rats (*n* = 36).

The serum concentrations of 25(OH)D and 1,25(OH)_2_D are shown in Table 3. No significant differences in either 25(OH)D or 1,25(OH)_2_D were found between pups at the two age groups (PND21 and PND30) in both control and Pb-exposed animals. At both PND21 and PND30, Pb exposure significantly decreased serum 25(OH)D compared to the control group (*p* = 0.0001 and *p* = 0.01, for PND21 and PND30, respectively). Serum 1,25(OH)_2_D was decreased significantly in the Pb exposed rats at PND21 (*p* = 0.04) but not at PND30 (*p* = 0.18). The association between BPbL and vitamin D metabolites is shown in Figure 1. Regression analysis showed significant negative associations between BPbL and 25(OH)D in both PND21 and PND30 groups were found (PND21; β = −0.64, *R*^2^ = 0.47, *p* < 0.0001 and PND30; β= −0.78, *R*^2^ = 0.25, *p* < 0.01). BPbL was negatively associated with 1,25(OH)_2_D levels in the PND21 pups (β = −0.02, *R*^2^ = 0.22, *p* < 0.05) but not in the PND30 pups (β = −0.02, *R*^2^ = 0.05, *p* > 0.05).

### 3.2. Effects of Pb Exposure on 1-α-Hydroxylase Expression and Distribution in Cerebrum and Kidney

**Figure 2:**
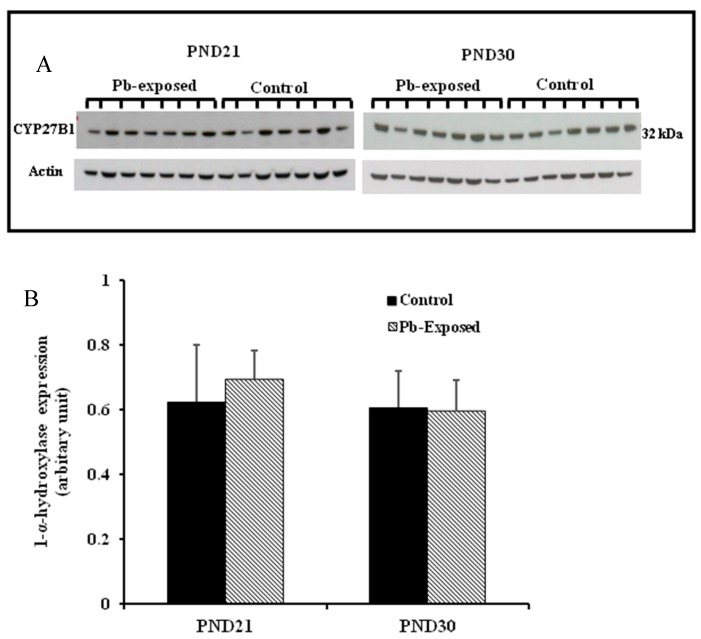
Effect of Pb exposure on the expression of 1-α-hydroxylase in the cerebral hemispheres of Wistar rat pups. (**A**) Western blot analysis of cerebral tissue of control and Pb-exposed rats with 1-α-hydroxylase antibody at PND21 and PND30: 20 µg of the lysate was resolved on 10% SDS-PAGE and immunoblotted with antibody to 1-α-hydroxylase (CYP27B1). For loading control, the same membranes were stripped and re-probed with anti-actin antibody; (**B**) Quantification of Western blot bands at 32 kDa for 1-α-hydroxylase on the blots (shown in A): Blots (both CYP27B1 and actin) were scanned and the band densities quantified with Syngene gene tool software. Signal for this enzyme was normalized to signal for β-actin (for a loading control). Data are presented as Mean ± SD (*n* = 7).

**Figure 3:**
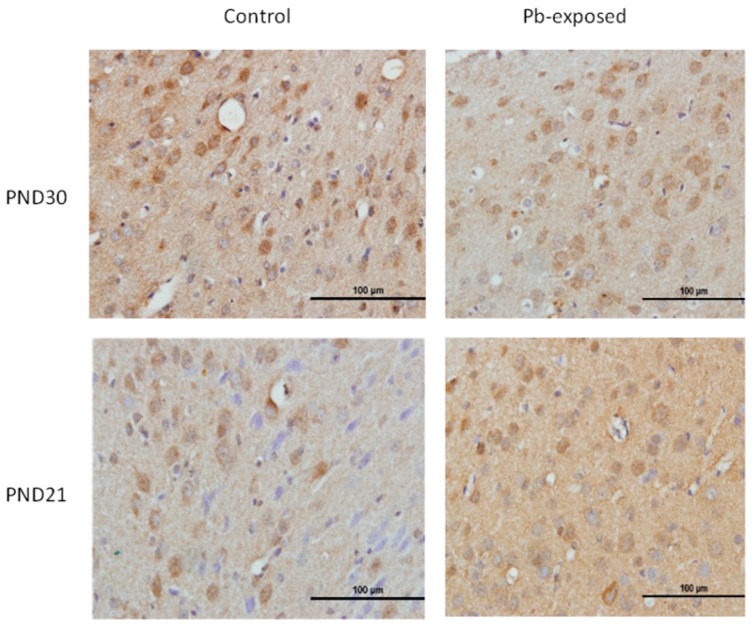
Immunohistochemistry photomicrographs showing the effects of Pb exposure on the expression of 1-α-hydroxylase in cerebral cortex of PND21 and PND30 rats. Sections (*n* = 3) counterstained with Mayer’s hematoxylin were photographed with oil immersion lens.

**Figure 4:**
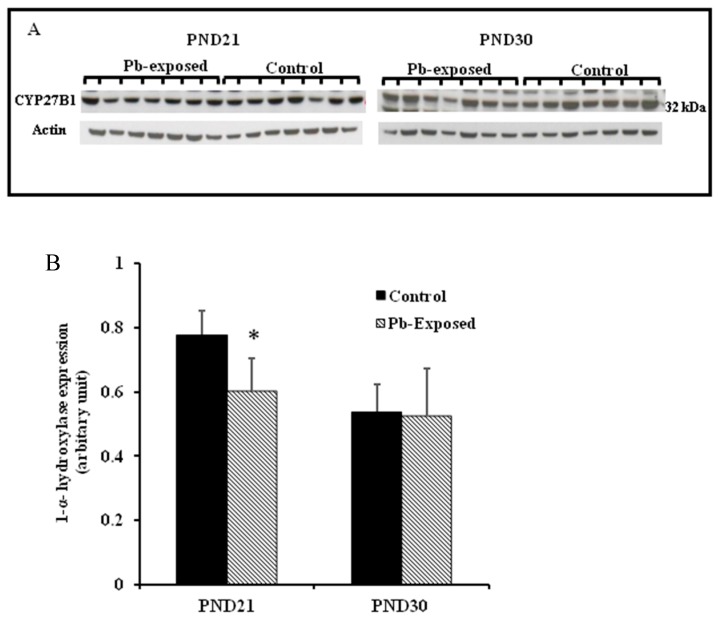
Effect of Pb exposure on the expression of 1-α-hydroxylase in the kidney of Wistar rat pups: (**A**) Western blot analysis of kidney tissue of control and Pb-exposed rats with 1-α-hydroxylase (CYP27B1) antibody at PND21 and PND30: 20 µg of kidney lysate was resolved on 10% SDS-PAGE and immunoblotted with antibody to CYP27B1. For loading control, the same membranes were stripped and re-probed with anti-actin antibody; (**B**) Quantification of Western blot bands at 32 kDa for CYP27B1 on the blots (shown in A): Blots (both CYP27B1 and actin) were scanned and the band densities quantified with Syngene gene tool software. Signal for this enzyme was normalized to signal for β-actin (for a loading control). Data are presented as Mean ± SD (*n* = 7). * Significantly different from control (*p* < 0.05).

**Figure 5:**
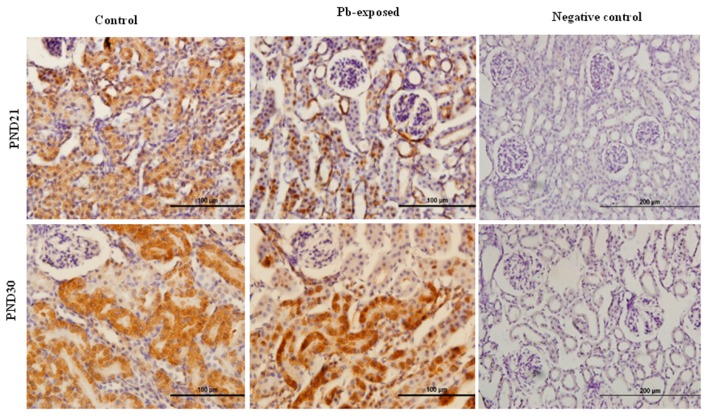
Immunohistochemistry photomicrographs showing the effects of Pb exposure on the expression of 1-α-hydroxylase in kidney of both control and Pb-exposed at PND21 and PND30 rats. Sections (*n* = 3) were counterstained with Mayer’s hematoxylin.

The expression of brain 1-α-hydroxylase (CYP27B1) was not significantly affected by Pb-exposure either at PND21 or PND30 (Figure 2). CYP27B1 was visualized in the cerebral cortex in both control and Pb-exposed pups at both age groups (Figure 3). In the kidney, Pb exposure resulted in a significant decrease of CYP27B1 level (by 22%) in the PND21 group (*p* = 0.01), whereas in the PND30 group, Pb exposure had no effect on the expression of renal CYP27B1 (Figure 4). The immunohistochemistry of kidney tissue (Figure 5) showed that the expression of CYP27B1was more pronounced in the proximal convoluted tubules and was largely cytoplasmic. Parallel to the Western blot results, fewer CYP27B1 immunoreactive cells were visualized by immunohistochemistry in the kidneys of PND21 pups but not in the PND30 Pb-exposed pups as compared to their respective controls.

### 3.3. Effects of Pb Exposure on 25-Hydroxylase Expression in Liver

**Figure 6:**
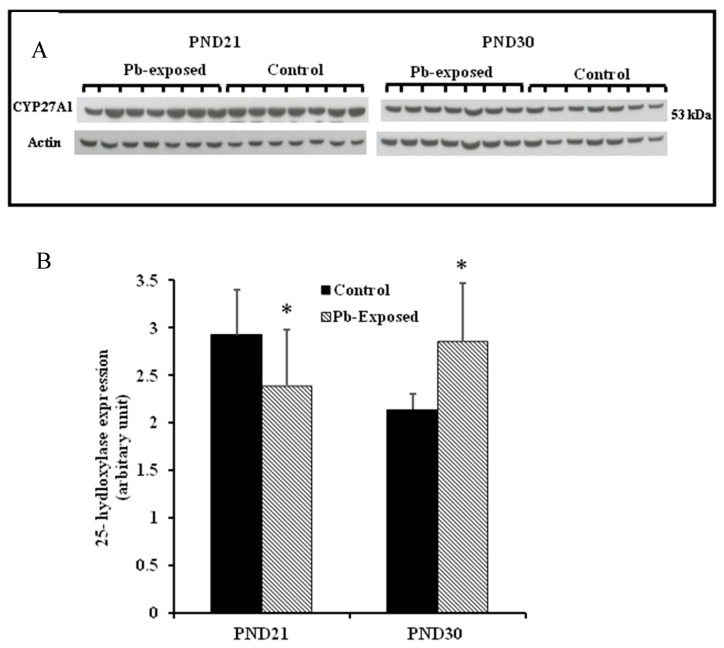
Effect of Pb exposure on the expression of 25-hydroxylase in the liver of Wistar rat pups: (A) Western blot analysis of liver tissue of control and Pb-exposed rats with 25-hydroxylase (CYP27A1) antibody at PND21 and PND30: 20 µg of liver lysate was resolved on 10% SDS-PAGE and immunoblotted with antibody to CYP27A1. For loading control, the same membranes were stripped and re-probed with anti-actin antibody; (**B**) Quantification of Western blot bands at 53 kDa for 25-hydroxylase on the blots (shown in A): Blots (both CYP27A1 and actin) were scanned and the band densities quantified with Syngene gene tool software. Signal for this enzyme was normalized to signal for β-actin (loading control). Data are presented as Mean ± SD (*n* = 7). * Significantly different from control (*p* < 0.05).

**Figure 7:**
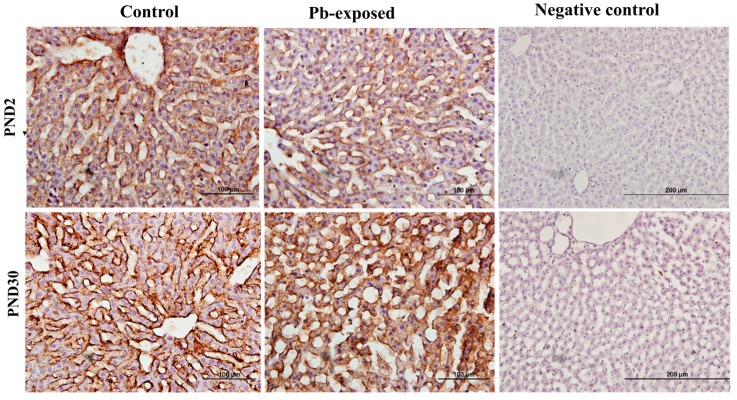
Immunohistochemistry photomicrographs showing the effect of Pb exposure on 25-hydroxylase expression in liver of both control and Pb-exposed rats at PND21 and PND30. Sections (*n* = 3) were counterstained with Mayer’s hematoxylin.

The expression of hepatic 25-hydroxylase (CYP27A1) in response to Pb exposure was significantly reduced (21%) in PND21 rat pups (*p* = 0.04), whereas, in PND30, it was significantly increased by 33% (*p* = 0.02) (Figure 6). The immunohistochemistry of liver tissue showed that CYP27A1 was expressed across the organ in both control and Pb-exposed rats at both PND21 and PND30 (Figure 7).

### 3.4. Effects of Pb Exposure on VDR Expression in Cerebrum

**Figure 8:**
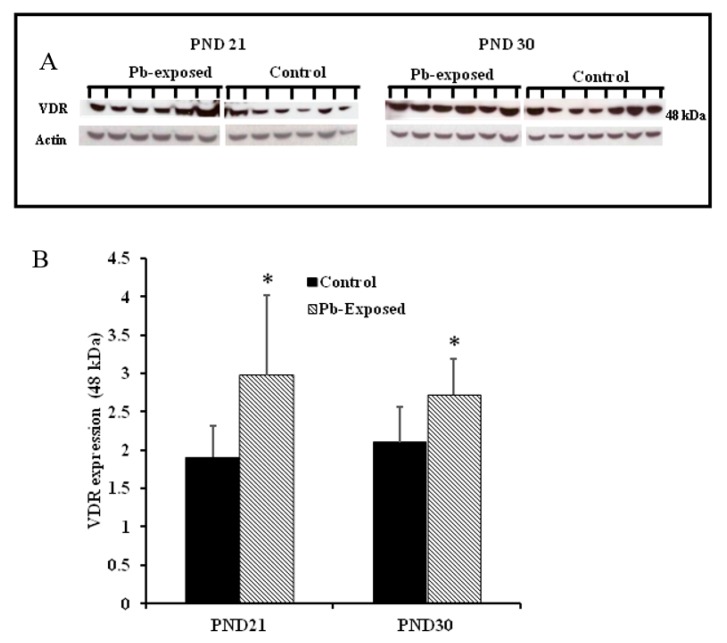
Effect of Pb exposure on VDR expression in the brain of Wistar rat pups: (**A**) Western blot analysis of brain tissue of control and Pb-exposed rats with VDR antibody at PND21 and PND30: 20 µg of brain lysate was resolved on 10% SDS-PAGE and immunoblotted with antibody to VDR. For loading control, the same membranes were stripped and re-probed with anti-actin antibody; (**B**) Quantification of Western blot bands at 48 kDa for VDR on the blots (shown in A): Blots (both VDR and actin) were scanned and the band densities quantified with Syngene gene tool software. Signal for VDR was normalized to signal for β-actin (loading control). Data are presented as Mean ± SD (*n* = 7). * Significantly different from control (*p* < 0.05).

**Figure 9:**
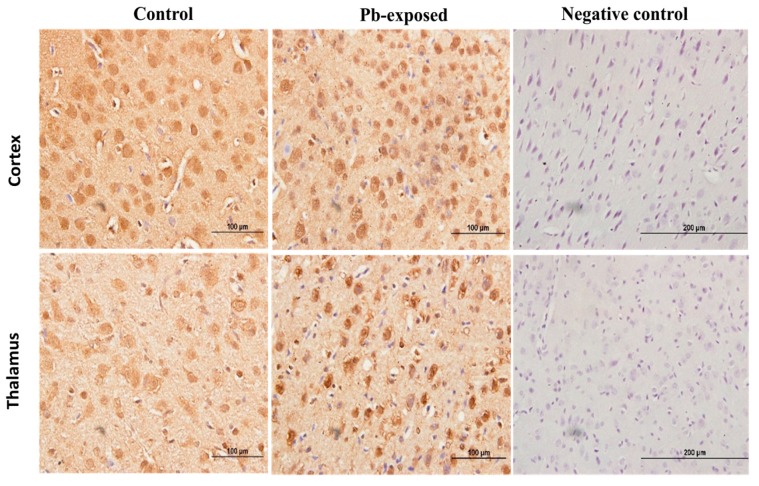
Immunohistochemistry photomicrographs showing the effect of Pb exposure on the expression of VDR in cerebrum of both control and Pb-exposed PND21 rats. Sections (*n* = 3) were counterstained with Mayer’s hematoxylin. CR, cortex; TH, thalamus.

**Figure 10:**
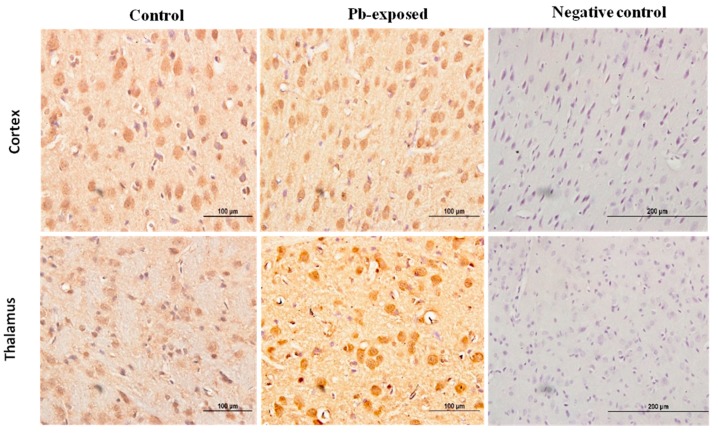
Immunohistochemistry photomicrographs showing the effect of Pb exposure on the expression of VDR in cerebrum of both control and Pb-exposed PND30 rats. Sections (*n* = 3) were counterstained with Mayer’s hematoxylin. CR, cortex; TH, thalamus.

Pb exposure significantly increased VDR expression in the cerebral hemispheres by 20% at PND21 (*p* = 0.03) and by 29% at PND30 (*p* = 0.02) (Figure 8). The immunohistochemistry of VDR in the Pb-exposed rats was consistent with Western blot results. More neurons in the Pb-exposed rats were immunoreactive for VDR in comparison to their respective controls (Figure 9 and Figure 10). The localization of VDR was restricted to the nucleus of brain cells and was observed in the cerebral cortex as well as the thalamus in both control and Pb-exposed rats at both age groups.

## 4. Discussion

The present study aimed to investigate whether Pb interferes with vitamin D metabolism by affecting the expression of its metabolizing enzymes (25-hydroxylase and 1-α-hydroxylase) and VDR in Wistar rats. To our knowledge, this is the first experimental study that examined the effects of Pb on serum vitamin D concentrations (both 25(OH)D and 1,25(OH)_2_D), expression of 25-hydroxylase and 1-α-hydroxylase and VDR in organs that are involved in vitamin D metabolism; both quantitatively and qualitatively employing Western blotting and immunohistochemistry.

### 4.1. Model Appropriateness and Pb Exposure Protocol

The daily oral administration of Pb in drinking water mimics the environmental Pb exposure to which children are generally exposed. Measuring BPbL is the most sensitive biomarker for reflecting current exposure to Pb. We used Wistar rat model since it has been extensively used to detect the Pb-induced toxicity in brain [13,33,34,35], liver [36,37,38] and kidneys [39,40,41]. Our Pb exposure protocol did not significantly affect total body weight or organ weights and these results correspond with the findings from previous studies in rats [35,42,43,44]. The Pb exposure protocol used in the present study significantly increased BPbLs at both PND21 and PND30 in the Pb exposed animals compared to their corresponding controls. These results are comparable with previously reported data using similar Pb exposure protocol (0.2% Pb acetate) [13,34,35,38].

### 4.2. Effects of Pb on Serum VD Metabolism

Rat pups were fed normal EURodent Diet 14% (Cat. No. 5LF2, LabDiet, St. Louis, MO, USA) which contained 1.0 IU of vitamin D_3_ per gram of food. The estimated average food intake for a PND21 rat is 15 g/day, whereas, it is 30 g/day for a PND30 rat [45]. Based on this estimated food intake, the average intake was 15 IU/day and 30 IU/day of vitamin D_3_ for the PND21 and PND30 pups, respectively. Although the actual food intake was not measured in this study, it is assumed that the food intake of control and Pb exposed rats was similar, as no weight changes were observed. Therefore, it could be inferred that the apparent difference in serum vitamin D concentrations between control and Pb-exposed rats was not due to the difference in vitamin D intake. Serum 25(OH)D concentrations in the control pups (Table 3) are within the normal range of rat of this age group [46,47]. Similarly, serum 1,25(OH)_2_D in the control pups were comparable with previously reported levels of this metabolite in control rats with similar vitamin D intake [45,46,47,48], indicating that our control pups were vitamin D sufficient. Furthermore, the lack of significant differences in the concentrations of 25(OH)D and 1,25(OH)_2_D between control pups of PND21 and PND30 and between Pb-exposed pups of PND21 and PND30 indicate that vitamin D levels during these developmental stages are age-independent. Interaction between vitamin D and serum concentration and/or dietary intake of essential divalent metal ions (Ca, Cu, Fe and Zn) and toxic heavy metals (Pb and Cd) has been reported [27]. In particular, the effect of high BPbL on these metal ions is well known. We have observed a 30–35% reduction in the blood concentrations of Cu, Mn and Zn in a similar protocol of Pb exposure in rats (unpublished); however, the levels of these metals were still within the physiological range. Furthermore, changes in the 25(OH)D concentration reported in this study are unlikely to affect Ca levels, as we have previously reported [32].

Our results showed that early postnatal Pb exposure of rats significantly decreased serum 25(OH)D levels at both PND21 and PND30 (Table 2). This was further confirmed with the strong negative association between BPbLs and serum 25(OH)D levels (PND21; β = −0.64, *R*^2^ = 0.47, *p* < 0.0001 and PND30; β = −0.78, *R*^2^ = 0.25, *p* < 0.01) in all rat pups of the same age (*n* = 31) irrespective of Pb exposure group. Few epidemiological studies have reported similar significant negative associations between Pb and serum 25(OH)D among women [49], Pb-exposed workers [20,21] and children [22,25]. Kersey et al. [50] have found negative but statistically insignificant, association between BPbls and serum 25(OH)D levels in healthy toddlers and children under 6 years of age. The 25(OH)D deficiency induced by Pb may indicate that Pb exposure interfered with hepatic 25-hydroxylase that is exclusively responsible for converting vitamin D into its major circulating form; 25(OH)D. Furthermore, the effect of Pb on serum 25(OH)D levels in rats shown in the present study and in the reported studies in humans suggests that the effect of Pb on 25(OH)D is not species specific.

We showed that the expression of hepatic CYP27A1 was significantly decreased by 21% at PND21, whereas, at PND30 it was significantly increased by 33%. These results do not parallel serum 25(OH)D changes that we observed. This apparent discrepancy in the effect of Pb on the expression of CYP27A1 in the liver suggest that Pb-induced effects on liver are dependent on the developmental stage of the animal. The significant increase in CYP27A1 at PND30 may also be a compensatory mechanism for the decrease in serum 25(OH)D levels. The increased level of CYP27A1 but the decreased level of 25(OH)D may also suggest that although the synthesis of the enzyme is increased in response to the low level of circulating 25(OH)D level, its activity may be affected by Pb. The enzyme quantity reflects both its synthesis and degradation and therefore higher levels of the enzyme expression do not necessarily imply that they are also highly active [51]. This apparent discordance in the PND30 rats may also be explained by the activation of 24-hydroxylase which is a catabolizing enzyme for both 25(OH)D and 1,25(OH)_2_D. However, the expression of this enzyme was not measured in this study and this remains speculative. The immunohistochemistry results are parallel with the Western blot results as the photomicrographs of liver sections showed more pronounced expression of CYP27A1 in liver sections from PND30 rats compared to PND21 rats. Previous studies have shown that hepatic CYP27A1localized more in mitochondria and microsomes [52,53].

In addition, we found that Pb exposure significantly decreased serum 1,25(OH)_2_D levels only in the PND21 rats (Table 3). This effect was also confirmed by a negative association between BPbLs and serum 1,25(OH)_2_D levels among this age group (β = −0.02, *R*^2^ = 0.22, *p* < 0.05, *n* = 28). Similar reduction in serum 1,25(OH)_2_D serum levels was reported by Smith et al., (1981) in Pb-exposed rats. In accordance with our results, previous studies have reported reduced serum 1,25(OH)_2_D levels in Pb exposed children [23,24] and female smelter workers [54]. They attributed this reduction to the inhibition of renal tubular CYP27B1 and therefore impaired renal biosynthesis of 1,25(OH)_2_D. In contrast, other studies have shown an increase in 1,25(OH)_2_D levels in Pb exposed rats, which has been explained as a compensatory mechanism to help normalize circulating calcium levels [55]. The observation that we did not find significant decrease in circulating 1,25(OH)_2_D levels in the Pb exposed rats at PND30 despite significant decrease in serum 25(OH)D levels may also be explained by such mechanism. Fullmer [56] has argued that Pb-induced effects on serum 1,25(OH)_2_D is Ca-dependent. He found that severe Ca deficiency was linked with low serum levels of 1,25(OH)_2_D and the levels were higher with less severe Ca deficiency.

Although CYP27B1 is mainly expressed in the kidney [3,52], it is also expressed in several other organs and tissues including the brain, activated cells of the immune system and the skin [3,4,52,53,57]. We selected kidney and the brain for the expression of this enzyme. The rationale is that kidney is the main site for the expression of this enzyme and it is involved in the calcemic effects of VD. As Pb is neurotoxic and is known to impair learning and memory, we were interested to investigate if the Pb-induced neurotoxicity might involve the dysregulation of brain CYP27B1. We found that the expression of CYP27B1 in renal tissue was significantly decreased by 22% only in PND21 Pb-exposed rats (Figure 5B), whereas it was not affected in PND30 rats. These results are perfectly in line with the serum 1,25(OH)_2_D, which was significantly decreased in PND21 Pb-exposed rats but not in PND30 Pb-exposed rats. In the brain tissue, on the other hand, the expression of this enzyme was not affected by Pb exposure at both age groups (Figure 5A). This apparent discrepancy in the effects of Pb in the kidney and brain CYP27B1 may suggest that these two enzymes are regulated by different mechanisms. The immunohistochemical localization of CYP27B1 was cytoplasmic and was present markedly in the proximal convoluted tubules in kidneys, as is well known from many previous studies [3,52,53].

### 4.3. Effects of Pb on VDR

VDRs are abundantly expressed in the brain. The expression of CYP27B, together with the expression of VDR in the brain, suggests that vitamin D is involved in brain development and function. Several studies including some from our laboratory have shown that VVD impairs learning and/or memory [32,58,59,60]. To explore the involvement of vitamin D signaling in Pb-induced neurotoxicity, we investigated the effect of Pb exposure on VDR expression in the brain. VDR has been shown to be widely distributed in rat and human brain and its pattern of distribution in the human brain was found to be similar to that of the rat [3,4,57]. Our results showed that Pb exposure caused significant increase of VDR expression at both age groups (Figure 5D). The biological actions of 1,25(OH)_2_D are mediated by its interaction with VDR [53]. This up-regulation of VDR expression in Pb-exposed rats at both PND21 and PND30 seems to be a response to the low circulating levels of 25(OH)D, which was decreased by Pb exposure in both age groups. VDR level is also thought to be a determinant of cellular responsiveness to VD [61]. The VDR gene polymorphism has been found to increase or decrease human susceptibility to Pb poisoning [62] and different VDR genotypes have been associated with different Pb-induced toxic effects [62,63,64,65,66,67]. Immunohistochemical results have shown that the VDR expression was restricted to the nucleus and localized mainly to brain cortex and thalamus (Figure 9 and Figure 10) and these results are in agreement with previously reported data [4].

### 4.4. Study Limitations

There are few limitations in this study. First, a rat model was used in this study. Although the expressions of vitamin D metabolizing enzymes and VDR in rats are similar to that in humans, the effect of Pb on vitamin D metabolism may not be necessarily the same. As such, studies could not be conducted in humans due to ethical issues, using animal models was the only choice for conducting this study. Second, we did not study the male and female pups separately to investigate the gender-dependent effect of Pb exposure on vitamin D metabolism. Third, we studied the effects of Pb on the level of vitamin D activating enzyme in different tissue but did not study the effects of Pb on enzyme activity. Fourth, the catabolizing enzyme 24-hydroxlase is also involved in regulating serum concentrations of 25(OH)D and 1,25(OH)_2_D but the effect of Pb on this enzyme was not studied and thus should be included in future studies. Furthermore, this study did not address the mechanism(s) leading to these effects of Pb on the expression of vitamin D metabolizing enzymes and VDR. Further studies are warranted to investigate the physiological implications of these findings and the biochemical mechanism(s) by which Pb affects the expression of vitamin D metabolizing enzymes and VDR.

## 5. Conclusions

In conclusion, this study indicates that the influence of Pb on the expression of vitamin D activating enzymes is tissue-specific and depends on the developmental stage of the animal. The role of vitamin D in brain development and function is well-established. Our findings suggest a novel mechanism of Pb-induced neurotoxicity. Based on our findings, we propose that Pb-induced reduction in serum 25(OH)D is involved in Pb-induced impairment of learning and memory. Further research, with properly designed experimental studies, is needed to test this hypothesis. In addition, further research is needed to elucidate the biochemical mechanism/(s) of these effects on the expression of CYP27A1 in liver and CYP27B in kidney and their physiological relevance by studying the activity of these enzymes. Furthermore, the mechanism by which Pb increases the expression of VDR and the implications of this increased expression of VDR in brain development and function need to be investigated in future research.

## Figures and Tables

**Table 1:** List of Primary Antibodies.

Primary Antibody	Tissue	Colonality	Dilution	Source
Anti-CYP27B1 (1-α-hydroxylase)	Brain, kidney	polyclonal	1:1000 for WB 1:100 for IHC	USCN Life Sciences Inc., Wuhan, China
Anti-CYP27A1 (25-hydroxylase)	Liver	monoclonal	1:1000 for WB 1:100 for IHC	Abcam, Cambridge, MA, USA
VDR antibody	Brain	monoclonal	1:1000 for WB and IHC	Aviva systems biology, San Diego, CA, USA
Anti-β-actin	All the above tissues	monoclonal	1:2000	Sigma-Aldrich, St. Louis, MO, USA

**Table 2:** Mean (±SD) body and tissue weight (g) of control and Pb-exposed Wistar rat pups at PND21 and PND30.

	PND21		PND30	
	Control	Pb-Exposed	Control	Pb-Exposed
	(*n* = 20)	(*n* = 20)	(*n* = 19)	(*n* = 19)
Body weight	35.0 ± 3.2	33.0 ± 7.8	71.1 ± 7.1	71.6 ± 13.6
Brain weight	1.3 ± 0.1	1.3 ± 0.1	1.4 ± 0.2	1.3 ± 0.2
Liver weight	1.2 ± 0.2	1.2 ± 0.3	1.9 ± 0.2	1.8 ± 0.3
Kidney weight	0.4 ± 0.1	0.4 ± 0.1	0.8 ± 0.1	0.9 ± 0.2

SD: standard deviation; PND: postnatal day.

**Table 3:** Blood lead and serum vitamin D metabolites (Mean ± SD) in control and Pb-exposed Wistar rat pups at PND21 and PND30.

	PND21		PND30	
	Control (*n* = 19)	Pb-Exposed (*n* = 20)	Control (*n* = 19)	Pb-Exposed (*n* = 20)
Pb (μg/dL)	2.2 ± 0.7	12.4 ± 3.3 **	3.3 ± 1.7	22.7 ± 6.0 **
25(OH)D (nmol/L)	32.2 ± 7.5	23.3 ± 5.7 *	32.2 ± 6.3	27.0 ± 6.4 *
1,25(OH)_2_D (pmol/L)	428.8 ± 132.8	361.5 ± 83.6 *	416.9 ± 129.1	379.7 ± 108.1

* Significantly different from respective control (*p* < 0.05). ** Significantly different from respective control (*p* < 0.000001).

## Abbreviations

Pb	lead
VDD	vitamin D deficiency
25(OH)D	25-Hydroxyvitamin D
1,25(OH)_2_D	1, 25-Dihydroxyvitamin D
VDR	Vitamin D receptor
CYP27A1	25-Hydroxylase
CYP27B1	1α-Hydroxylase
PTH	Parathyroid hormone
PND	Postnatal day
BPbL	Blood lead level
LC-MS/MS	Liquid chromatography-tandem mass spectrometry
ICP-OES	Inductively coupled plasma-optical emission spectrometer
